# Nanoparticulate Double-Heterojunction Photocatalysts Comprising TiO_2(Anatase)_/WO_3_/TiO_2(Rutile)_ with Enhanced Photocatalytic Activity toward the Degradation of Methyl Orange under Near-Ultraviolet and Visible Light

**DOI:** 10.1021/acsomega.0c06054

**Published:** 2021-04-28

**Authors:** José Alfonso Pinedo-Escobar, Junpeng Fan, Edgar Moctezuma, Christian Gomez-Solís, Cristina Jared Carrillo Martinez, Eduardo Gracia-Espino

**Affiliations:** † Unidad Académica de Ciencias Químicas, Universidad Autónoma de Zacatecas, Campus Universitario Siglo XXI, km. 6 Carr. Zacatecas-Guadalajara s/n Ejido La Escondida, Zacatecas 98160 Zacatecas, México; ‡ Department of Physics, Umeå University, Umeå 90187, Sweden; § Facultad de Ciencias Químicas, Universidad Aut́noma de San Luis Potosí, Av. Manuel Nava #6, San Luis Potosí 78290 San Luis Potosí, México; ∥ División de Ciencias e Ingenieŕa, Universidad de Guanajuato, León 37150, Guanajuato, México

## Abstract

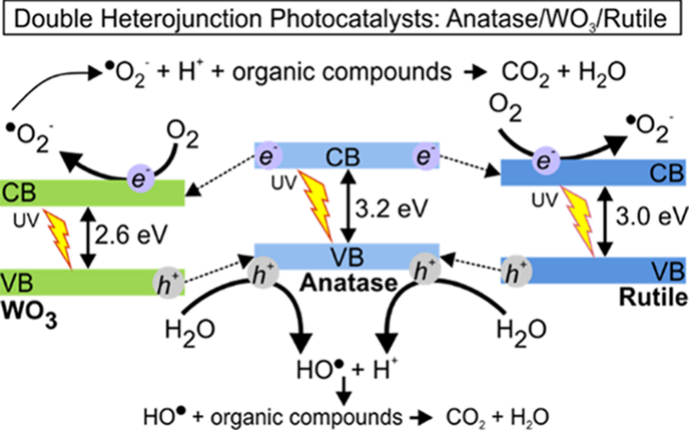

Nanoparticulate double-heterojunction photocatalysts comprising TiO_2(Anatase)_/WO_3_/TiO_2(Rutile)_ were produced by a sol–gel method. The resulting photocatalysts exhibit clear synergistic effects when tested toward the degradation of methyl orange under both UV and visible light. Kinetic studies indicate that the degradation rate on the best double-heterojunction photocatalyst (10 wt % WO_3_-TiO_2_) depends mainly on the amount of dye concentration, contrary to pure oxides in which the degradation rate is limited by diffusion-controlled processes. The synergistic effects were confirmed through systematic and careful studies including holes and OH radical formation, X-ray diffraction, electron microscopy, elemental analysis, UV–vis diffuse reflectance spectroscopy, and surface area analysis. Our results indicate that the successful formation of a double heterojunction in the TiO_2(Anatase)_/WO_3_/TiO_2(Rutile)_ system leads to enhanced photoactivity when compared to individual oxides and commercial TiO_2_ P25.

## 1 Introduction

Technological applications of heterogeneous photocatalysis have been successfully reported in water and air treatment,^1−5^ CO_2_ reduction,^6−8^ and hydrogen production.^9−12^ Commercial TiO_2_ P25 is one of the most widely used photocatalyst. It is recognized for its good catalytic activity, high stability, insolubility in water, and low cost.^9,13−15^ Part of the success of TiO_2_ P25 under UV irradiation lies on the natural heterojunction formed between rutile and anatase; the presence of this junction enables the separation of photogenerated electron–hole (e^–^–h^+^) pairs, decreasing their recombination. However, TiO_2_ P25 can only work efficiently under UV illumination given its large band gap^16−19^ and suffers from high recombination of the photogenerated e^–^–h^+^ pairs.^20−23^

As a result, several efforts have been made to tune the absorption range of TiO_2_ toward the visible region. A common approach is the addition of dopants, such as noble metals^5,19,24,25^ or some light elements (e.g., C, N, or S).^3,26−29^ Another relevant strategy is the formation of heterostructures comprising TiO_2_ P25 and narrow band gap semiconductors (e.g., WO_3_) with a more negative (positive) conduction (valence) band level.^15,21,30−35^ The latter allows an efficient transfer of photogenerated electrons/holes from the guest semiconductor to TiO_2_, significantly reducing the e^–^–h^+^ recombination.^13^ For this particular reason, TiO_2_-WO_3_ composites are widely used as photocatalysts,^36−38^ where the 2.8 eV band gap of WO_3_, in addition to the positions of its valence and conduction bands, allows an efficient transfer of photogenerated holes from WO_3_ to TiO_2_ and electrons from TiO_2_ to WO_3_. The latter is possible due to the small standard reduction potential (−0.03 eV) between W(VI) and W(V).^39^ Subsequently, WO_3_ can transfer electrons to adsorbed oxygen molecules on TiO_2_, resulting in enhanced photocatalytic degradation of organic molecules.^13^

Therefore, here, we report the creation of a double-heterojunction photocatalyst comprising TiO_2_ rutile, TiO_2_ anatase, and monoclinic WO_3_. The larger affinity of WO_3_ toward anatase is used to minimize anatase transformation to rutile, and in addition, the coupling of WO_3_ with the anatase/rutile system results in enhanced charge transfer of photogenerated electron and holes, reducing recombination rates and enhancing the production of hydroxyl radicals under both visible and UV light illumination. The TiO_2(Anatase)_/WO_3_/TiO_2(Rutile)_ double heterojunction was produced by using a simple sol–gel method. The nanocomposite containing 10% of WO_3_ in a mixture of anatase and rutile exhibited clear synergistic effects during the photocatalytic degradation of methyl orange (MO) in both UV and visible light. The morphology, elemental composition, kinetic parameters, and degradation mechanisms are investigated.

## 2 Experimental Section

### 2.1 Synthesis of WO_3_

The monoclinic phase of tungsten trioxide was obtained by dissolving 2 g of ammonium tungstate hydrate ((NH_4_)_10_(H_2_W_12_O_42_)·4H_2_O) in deionized (DI) water (85 mL) at 80 °C. Afterward, concentrated HNO_3_ (15 mL) was added dropwise, and the suspension was kept under constant reflux and agitation for 30 min. The suspension was then transferred to an ultrasonic bath (30 min). The resulting solids were filtered, washed with DI water, and dried (70 °C, 24 h). The obtained powder was ground and calcined (500 °C, 4 h) in air.

### 2.2 Synthesis of Anatase and Rutile TiO_2_

Anatase TiO_2_ was synthetized by the sol–gel method using titanium isopropoxide (4.5 mL) dissolved in 2-propanol (50 mL) and acetic acid (5 mL). The hydrolysis was initiated by adding DI water (5 mL) dropwise. The solution was then kept at 70 °C for 60 min under constant agitation and reflux. Afterward, the sample was dried at 100 °C for 24 h, ground, and finally calcined at 400 °C (4 h) in air. This material was labeled TiO_2(Anatase)_. TiO_2_ rutile was prepared by direct calcination of 6 g of TiO_2_ Evonik P25 at 800 °C for 4 h in air, labeled as TiO_2_ P25_(Rutile)_.

### 2.3 Synthesis of Double-Heterojunction Photocatalysts

Three double-heterojunction photocatalysts with varying contents of WO_3_ (10, 20, and 30 wt %) and TiO_2_ were prepared, denoted as 10%WO_3_-TiO_2_, 20%WO_3_-TiO_2_, and 30%WO_3_-TiO_2_, respectively. Initially, a solution containing titanium isopropoxide (4.5 mL), 2-propanol (50 mL), and acetic acid (5 mL) was prepared. In a separate container, WO_3_ and TiO_2_ P25 (1 g) were mixed with 2-propanol (40 mL) for 30 min using an ultrasonic probe (VXC 130, Sonics & Materials, Inc.). This suspension was later combined with the initial acid solution. The hydrolysis was initiated by adding DI water (5 mL) dropwise; the mixture was kept at 70 °C for 60 min under constant agitation and reflux. The samples were dried at 100 °C for 24 h, then ground, and calcined at 800 °C for 4 h under air with a heating rate of 5 °C min^–1^.

### 2.4 Materials Characterization

X-ray diffraction was performed using a Bruker D8 Advance diffractometer (Cu Kα, λ = 1.5406 Å; 40 kV and 40 mA), and a step size of 0.05°·s^–1^ was used. The JCPDS (Joint Committee on Powder Diffraction Standards) database was used to identify the crystalline phases. The proportion of the crystalline phases was evaluated using the software Materials Analysis Using Diffraction (MAUD). The Scherrer equation was used to evaluate the crystallite sizes, *L* = κλ/β cos(θ), where κ (0.89) is the Scherrer constant, λ is the wavelength of the X-ray radiation, and β is the full width at half maximum of the diffraction peak at 2θ.^40^ The materials’ morphology was analyzed using a scanning electronic microscope (Carl Zeiss Merlin) equipped with an energy dispersive X-ray spectroscopy (EDX) analyzer, and a thin gold coating was used to improve the conductivity of the samples. Transmission electron microscopy (TEM) images were obtained on an FEI Talos L 120C. X-ray photoelectron spectroscopy (XPS) was performed with a Kratos Axis Ultra DLD electron spectrometer (Al Kα line of 1486.6 eV); the XPS spectra were calibrated with C 1s = 284.4 eV (C–C sp^2^).^41,42^ The experimental core-level spectra were fitted by using Gaussian curves, and a Shirley background subtraction was applied in the fitting process. The band gap (*E*_g_) was determined with UV–vis diffuse reflectance spectroscopy (Thermo Scientific Evolution 600) and the Kubelka–Munk method.^43^ All samples were examined in the 200–800 nm range, and then, by plotting [*h*(*c*/λ)*f*(*R*)]^0.5^ vs *hc*/λ, the *E*_g_ can be found when extrapolating the slope to intercept the *x* axis. Here, *h* = 6.62607004 × 10^–34^ m^2^ kg s^–1^, *c* is the speed of light in m s^–1^, λ is the wavelength (m), and *f*(*R*) is the information provided by the UV–vis spectrophotometer. The specific surface area was evaluated by using the Brunauer–Emmett–Teller (BET) method using nitrogen physisorption with the Nova 2000e Quantachrome Instruments. All samples were degassed at 300 °C for 1 h before the analysis.

### 2.5 Determination of the Photocatalytic Activity

The degradation was carried out in a reacting system comprising an annular stainless steel cylinder with an internal mirror and four symmetrically distributed near-UV light lamps T-15 L (15 W, λ_max_ = 365 nm; emission spectra shown in Figure S1). A Pyrex glass cylindrical cell is located at the center of the photoreactor, and it has three access ports for sampling, feeding, and gas evacuation.^1,2^ The photocatalytic activity under visible light was investigated using four FL15AQ lamps (15 W) adapted with a polycarbonate filter to ensure that only visible light reached the suspension (Figure S2 depicts the FL15AQ lamp emission spectra with and without a UV filter). A reaction volume of 250 mL, 0.5 g of photocatalyst, and 0.06 mM initial MO concentration were used during the degradation reactions. The total reaction time of 6 h was used, with a constant oxygen flow (100 mL min^–1^) and agitation. The reaction was monitored by taking a sample every hour, centrifuged, and analyzed using UV–vis spectroscopy.

### 2.6 Determination of Holes in the Photocatalysts’ Valence Band

The hole formation was investigated using the iodine ion (I^–^) reaction as shown in eq 1, where the I^–^/ pair has a potential equal to +0.536 V, which is smaller than the valence band potentials for WO_3_ (+2.9 V) and TiO_2_ (+2.7 V).
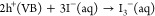
1

The  ion was monitored using UV–vis spectroscopy utilizing its two absorption peaks at 286 and 345 nm. The experiments were carried out by using an aqueous solution of potassium iodine (0.01 M, 250 mL) containing 0.5 g of photocatalyst. The suspension was kept in the dark for 20 min to reach the adsorption–desorption equilibrium. Afterward, all near-UV lamps were switched on, and samples were collected every 30 min. The analysis was performed using a UV–vis spectrophotometer (2401PC Shimadzu).

### 2.7 Determination of Hydroxyl Radicals

The formation of ^·^OH radicals was monitored by their oxidation with terephthalic acid where the produced 2-hydroxy-terephthalic acid can be quantified by using its fluorescence signal at 426 nm.^30^ In these experiments, a solution containing NaOH (0.01 M), terephthalic acid (20 ppm), and a photocatalyst (0.5 g) was prepared. The mixture was kept under dark conditions for 20 min to achieve an adsorption–desorption equilibrium. Afterward, all four near-UV lamps were switched on, and samples were extracted every 15 min in the first hour and then at 90 and 120 min. A Cary Eclipse fluorescence spectrophotometer was used to analyze the samples using an excitation wavelength of 315 nm, an emission range of 350–600 nm, a slit of 2.5, low voltage, and low scan.

## 3 Results and Discussion

### 3.1 Photocatalyst Characteristics: Crystal Phase, Band Gap, and Morphology

**Figure 1:**
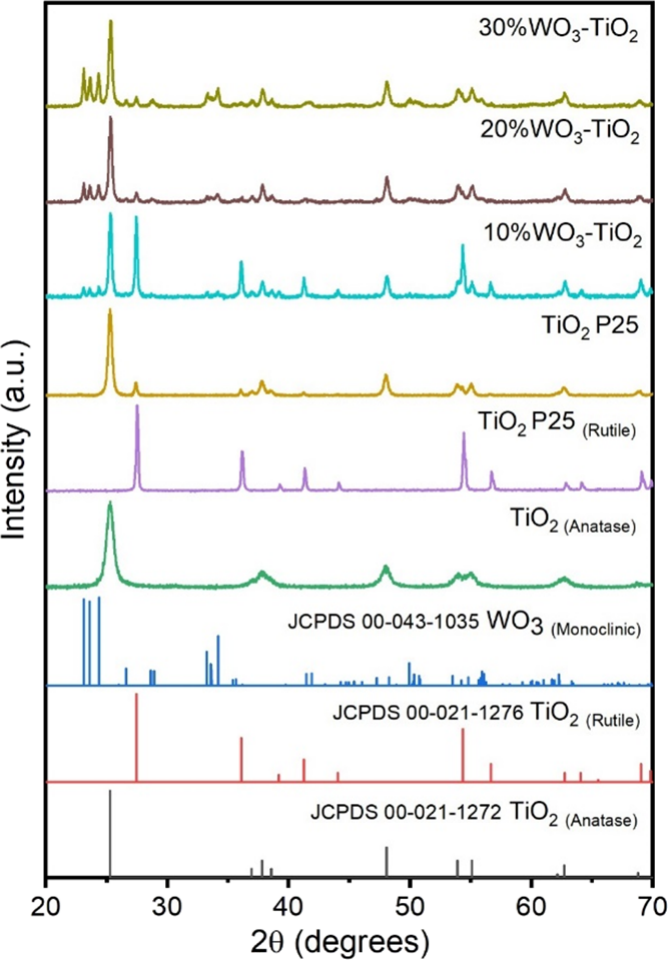
X-ray diffractograms of individual oxides and the three double-heterojunction photocatalysts. The reference JCPDS data are plotted for comparison.

The crystal structure, phase composition, and average crystallite size of the selected photocatalysts were investigated using powder X-ray diffraction. First, commercial TiO_2_ Evonik P25 (labeled as TiO_2_ P25) comprises both anatase and rutile phases (see Figure 1), with an estimated composition of 84.2% anatase and 15.8% rutile and average crystallite sizes of 28 and 167 nm for anatase and rutile, respectively. The subsequent calcination of TiO_2_ P25 at 800 °C in air results in the transformation of the anatase phase into rutile (JCPDS 00-021-1272) with an average crystallite size of 290 nm; this material was labeled as TiO_2_ P25_(Rutile)_. The TiO_2_ synthetized by the sol–gel method and calcined at 400 °C exhibited a pure anatase phase (JCPDS 00-021-1272) with a crystallite size of 12 nm. Last, the produced WO_3_ had a monoclinic phase type (JCPDS 00-043-1035) with a crystallite size of 54 nm. For the case of the double-heterojunction TiO_2(Anatase)_/WO_3_/TiO_2(Rutile)_ photocatalysts, three different WO_3_-TiO_2_ mixtures were considered, namely, 10, 20, and 30 wt % WO_3_ (labeled as 10%WO_3_-TiO_2_, 20%WO_3_-TiO_2_, and 30%WO_3_-TiO_2_, respectively). All heterojunctions consisted of both TiO_2_ anatase and TiO_2_ rutile, as well as monoclinic WO_3_. The proportion of crystalline phases is shown in Table 1. The results indicate that increasing the amount of tungsten trioxide inhibits the transformation of anatase into rutile, typically at 600 °C. This observation is consistent with previous reports,^44,45^ where tungsten atoms strongly interact with the edge of TiO_2_ crystals, limiting the crystal growth and anatase-to-rutile transition. The latter indicates an intimate contact between the individual components of our WO_3_-TiO_2_ heterojunction.

**Table 1:** Composition of Crystal Phases in Double-Heterojunction Photocatalysts^a^

crystal phase	10% WO_3_	20% WO_3_	30% WO_3_
TiO_2_ rutile	45.6 (223 nm)	10.1	8.1
TiO_2_ anatase	48.6 (45 nm)	81.7 (39 nm)	76.7 (38 nm)
WO_3_ monoclinic	5.8	8.2	15.2

a The numbers in parentheses indicate the crystallite size.

**Figure 2:**
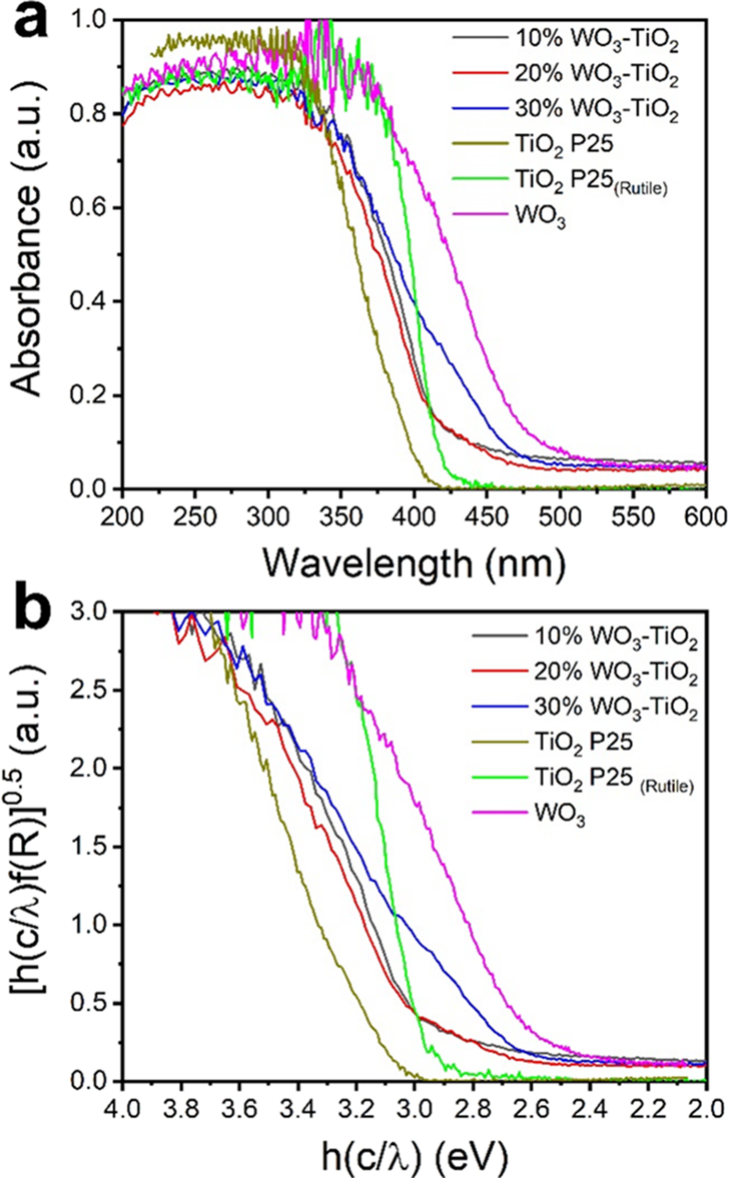
(a) Absorption spectra and (b) Kubelka–Munk plot of selected photocatalysts.

The band gap (*E*_g_) of all photocatalysts was evaluated using UV–vis diffuse reflectance spectroscopy. The resulting absorption spectra and Kubelka–Munk (Tauc) plots are shown in Figure 2. The *E*_g_ for pure oxides agrees with previous reports, as seen in Table 2. The commercial TiO_2_ P25 has an *E*_g_ of 3.2 eV, while the calcinated TiO_2_ P25_(Rutile)_ has a slight reduction to 3.0 eV; the reduced *E*_g_ is expected due to anatase phase transformation into rutile. The absorption spectra of all three WO_3_-TiO_2_ heterojunctions highlight their capacity to additionally absorb parts of the visible light, a desirable characteristic for photocatalysts since the solar spectrum contains predominantly visible light (45%).^40^ Both 10%WO_3_-TiO_2_ and 20%WO_3_-TiO_2_ photocatalysts have equal energy band gaps of 2.9 eV, which are smaller than 3 eV for pure TiO_2_ rutile. On the other hand, 30%WO_3_-TiO_2_ exhibits an even smaller *E*_g_ of 2.6 eV, an intermediate value between WO_3_ and rutile, highlighting the proper intermix of all three components. The latter indicates the suitability to modulate *E*_g_ by the addition of WO_3_.

**Table 2:** Band Gap of Heterojunctions and Individual Photocatalysts

photocatalyst	λ (nm)	*E*_g_ (eV) (this work)	*E*_g_ (eV)
10%WO_3_-TiO_2_	425	2.9	
20%WO_3_-TiO_2_	425	2.9	
30%WO_3_-TiO_2_	470	2.6	
TiO_2_ P25	397	3.2	3.37^46^
TiO_2_ P25_(Rutile)_	413	3.0	3.0^47^
WO_3_	485	2.6	2.5^48^

**Figure 3:**
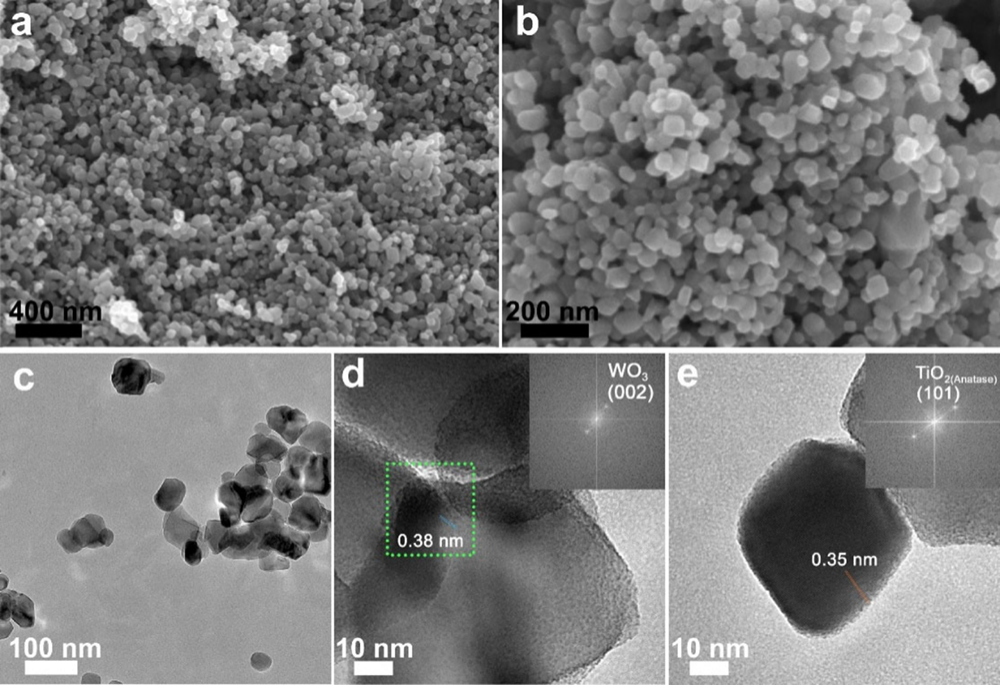
(a, b) SEM and (c–e) TEM micrographs of the double heterojunction 10%WO_3_-TiO_2_.

**Figure 4:**
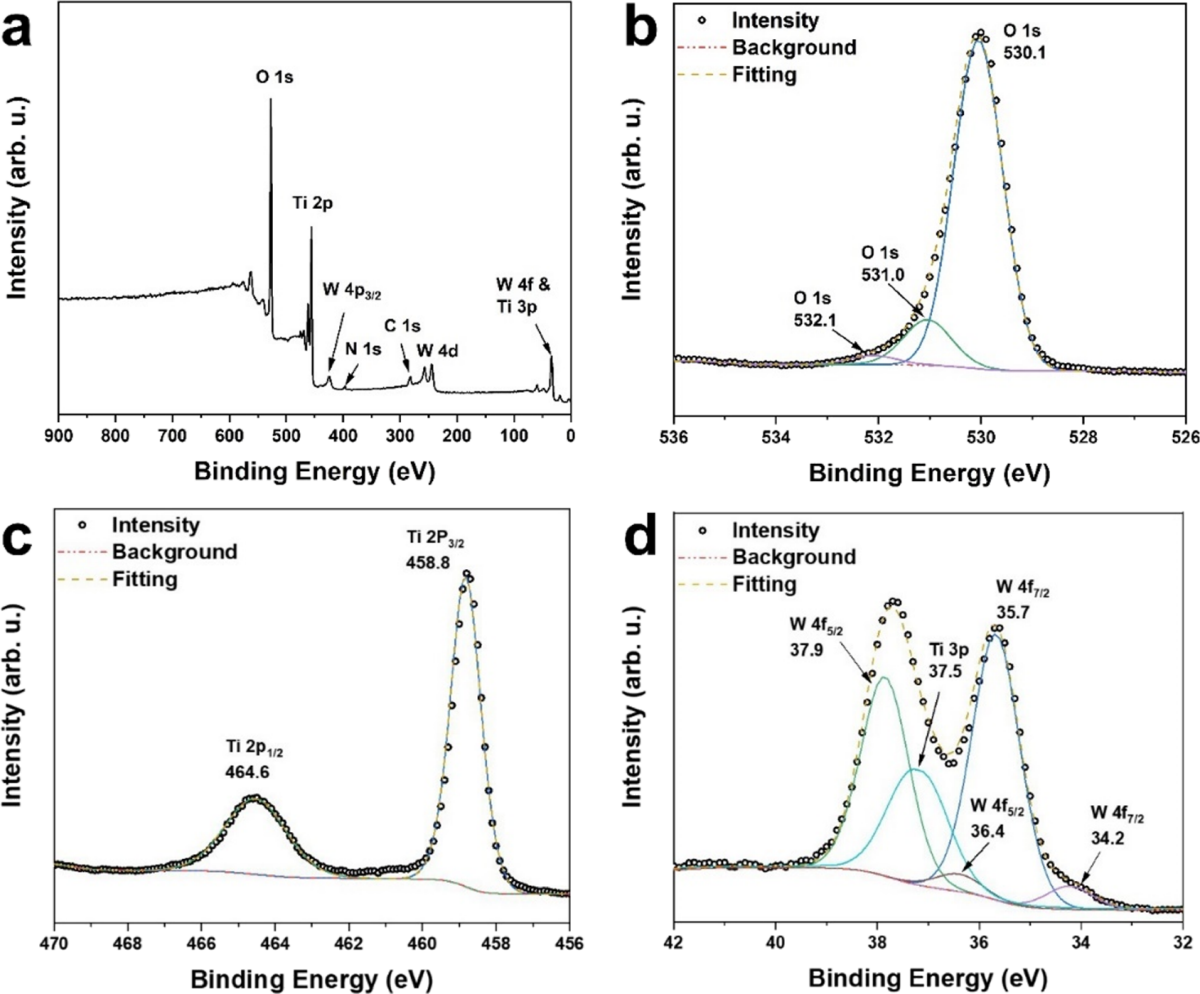
(a) Overall XPS spectra for 10%WO_3_-TiO_2_. High-resolution XPS of (b) O, (c) Ti, and (d) W.

We now focus on the 10%WO_3_-TiO_2_ nanocomposite to evaluate in detail the morphology and elemental composition. This double heterojunction showed the best photocatalytic activity, as we will discuss later. SEM studies (Figure S3a,b and Figure 3a,b) reveal a homogeneous nanoparticulated material with an average particle size of ∼50 nm. A closer inspection by TEM (Figure S3c–e and Figure 3c–e) shows that WO_3_ and TiO_2_ nanoparticles exhibit a thin layer of amorphous material. Although we could not identify the nature of such layer, similar amorphous layers have been previously observed in other WO_3_-TiO_2_ systems where it has been identified as tungsten oxides.^44^ In particular, Figure 3d,e shows two nanoparticles with clear crystalline planes corresponding to WO_3_ and TiO_2_ anatase, respectively. We further investigated the oxidation state of Ti and W by XPS studies. The survey spectrum of 10%WO_3_-TiO_2_ is displayed in Figure 4a, indicating only the existence of C, O, Ti, and W. The high-resolution spectra of O, Ti, and W are shown in Figure 4b–d, respectively. In Figure 4b, the deconvoluted XPS spectrum of O 1s includes three individual peaks. The main peak at 530.1 eV is assigned to the synthesized TiO_2_ and WO_3_, while the peak at 531.0 eV can be assigned to either substoichiometric WO*_x_*^49,50^ or hydroxyl groups adsorbed on the oxides’ surface.^51^ Another small feature at 532.1 eV can be attributed to the presence of oxygen-containing hydrocarbons,^52^ probably due to remnants from the synthesis process. Figure 4c shows only one doublet of Ti 2p at 458.8/464.6 eV with no other peaks, indicating that all Ti atoms have the same oxide state (Ti^4+^).^53,54^ On the other hand, the deconvolution of W 4f spectra yields two doublets, as shown in Figure 4d. The first one at 35.7/37.9 eV is attributed to W^6+^ in WO_3_,^55,56^ while the second one at 34.2/36.4 eV might be caused by the photoemission of W^5+^ present in substoichiometric WO*_x_* (2 < *x* < 3);^57,58^ this feature is consistent with the O 1s peak seen at 531.0 eV.

### 3.2 Photocatalytic Activity under Near-UV and Visible Light

**Figure 5:**
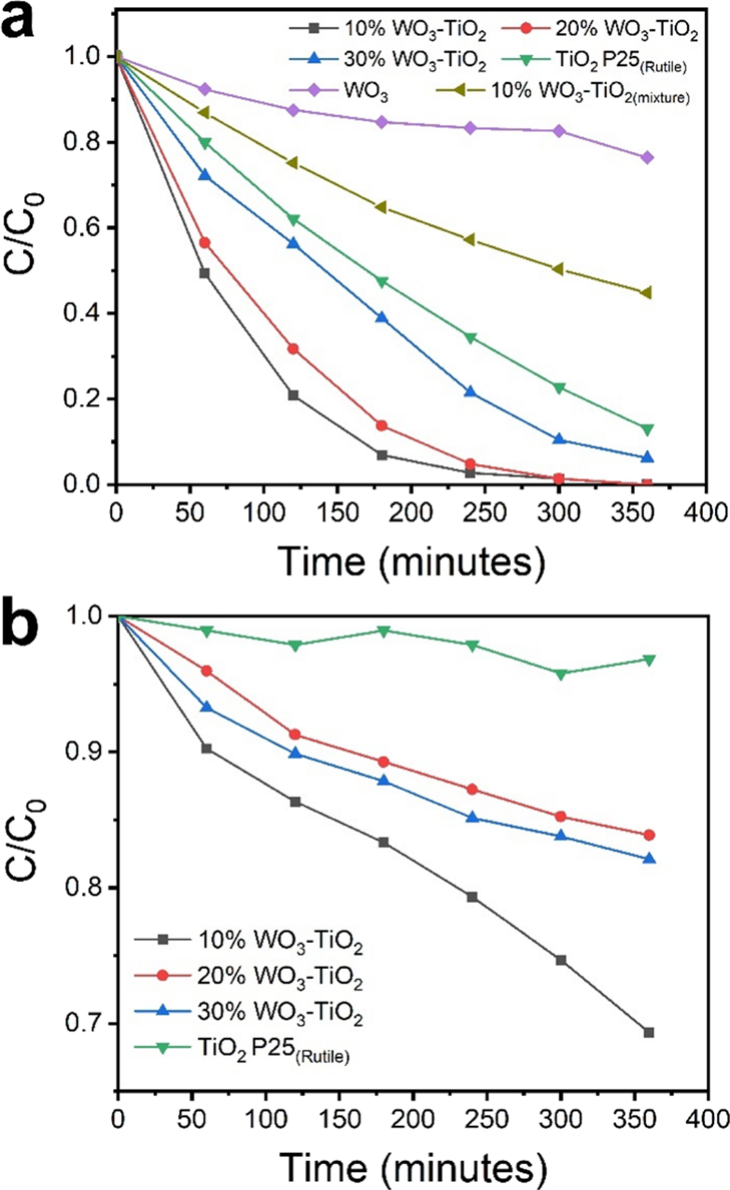
Degradation of methyl orange in aqueous solutions with (a) near-UV and (b) visible light illumination.

The degradation of methyl orange under near-UV and visible light irradiation was used to evaluate the photocatalytic activity; the results are shown in Figure 5a,b. All heterojunctions exhibited better MO degradation when compared to individual oxides, but in particular, both 10%WO_3_-TiO_2_ and 20%WO_3_-TiO_2_ photocatalysts exhibited the best photocatalytic activity with ∼99% degradation of MO after 300 min under near-UV irradiation, while 30%WO_3_-TiO_2_ only achieved ∼90% degradation during the same time. We also evaluated the MO degradation under similar conditions of a simple mixture of TiO_2_ P25 and WO_3_ (10 wt %) prepared by grinding the materials in a mortar, labeled as 10%WO_3_-TiO_2(mixture)_ in Figure 5a. This mixture only achieved ∼50% MO degradation after 300 min, clearly highlighting the importance of an intimate contact between the oxides achieved during the synthesis process. Figure 5b shows the results of MO degradation under visible light (436 nm). In this occasion, the 10%WO_3_-TiO_2_ photocatalyst clearly exhibited the best performance, achieving an MO degradation of ∼25% after 300 min, while the other heterojunctions only achieved 15–17%, and pure oxides showed negligible MO degradation. Although the 10%WO_3_-TiO_2_ photocatalyst exhibits a relatively large *E*_g_, large particle size, and low surface area (Table S1) when compared to other heterojunctions, the excellent photocatalytic activity indicates that the adequate distribution of the material and the crystal phase (rutile, 45.6 and 5.8% WO_3_) have a major role in achieving high catalytic activity. These results show the feasibility for a double-heterojunction system to reduce charge recombination, achieving a higher density of positive holes to produce hydroxyl radicals.

### 3.3 Kinetic Modeling

The kinetics of the photocatalytic degradation of MO when using near-UV illumination was examined with the first-order and modified Freundlich models.^59^ Here, the apparent reaction rate provides quantitative information regarding the MO degradation and mechanistic details. For the first-order model (see details in the Supporting Information), we assumed the Langmuir–Hinshelwood mechanism,^60^ in which the reaction rate, *r*, is given by
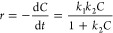
2

Here, *k*_1_ is the rate constant that includes parameters such as the maximum amount of compound adsorbed on the photocatalyst surface (see the Supporting Information for more details), *k*_2_ is the absorption constant, and *C* is the dye concentration. At a very low dye concentration, the term *k*_2_*C* ≪ 1, making the reaction rate apparently of first degree. We can now define the apparent reaction rate *r*_app_ and its apparent rate constant *k*_app_ (eq 3), which, after integration, results in eq 4.
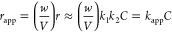
3
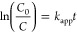
4where *w* is the photocatalyst mass, *v* is the volume of the reaction, and *C*_0_ and *C* are the initial dye concentration and the dye concentration after the photocatalytic degradation, respectively. On the other hand, the modified Freundlich kinetic model is characterized by eq 5,^61^
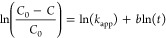
5

The constant in eq 5 has a similar meaning to that in eq 4. The kinetic parameters were estimated by doing a linear regression of the MO concentration in time (see Figures S4 and S5) and the experimental data in Figure 5a. The coefficient of determination *R*^2^ was then used to evaluate the quality of the fittings. The apparent reaction rates and *R*^2^ values are shown in Table 3. The obtained *R*^2^ values indicate that 10%WO_3_-TiO_2_ and 20%WO_3_-TiO_2_ heterojunctions follow the first-order model, which means that the degradation rate depends mainly on the amount of dye molecules in the solution. In contrast, other photocatalysts were better described by the modified Freundlich model, which describes a degradation mechanism controlled by ion-exchange and diffusion-controlled processes.^62^ The value of the apparent rate constant can be used to compare the photocatalytic activity, and as expected, the highest value of *k*_app_ was obtained for 10%WO_3_-TiO_2_ when compared to other photocatalysts (fitted by the same model). Therefore, independent of the model used, 10%WO_3_-TiO_2_ exhibits the best performance in agreement with the experimental observations.

**Table 3:** Kinetic Model and Apparent Reaction Rate

	first order		modified Freundlich	
photocatalyst	*k*_app_ × 10^–3^ (min^–1^)	*R*^2^	*k*_app_ × 10^–3^ (L g^–1^ min^–1^)	*R*^2^
10%WO_3_-TiO_2_	14.8	0.9958	96.8	0.9219
20%WO_3_-TiO_2_	14.1	0.9735	53.0	0.9717
30%WO_3_-TiO_2_	7.3	0.9576	13.0	0.9967
TiO_2_ P25	9.3	0.9533	11.5	0.9953
TiO_2_ P25_(Rutile)_	4.9	0.9871	6.6	0.9977
TiO_2(Anatase)_	6.0	0.9728	15.8	0.9973
WO_3_	0.6	0.8852	9.7	0.9594

### 3.4 Hole Formation and Determination of ·OH Radicals

**Figure 6:**
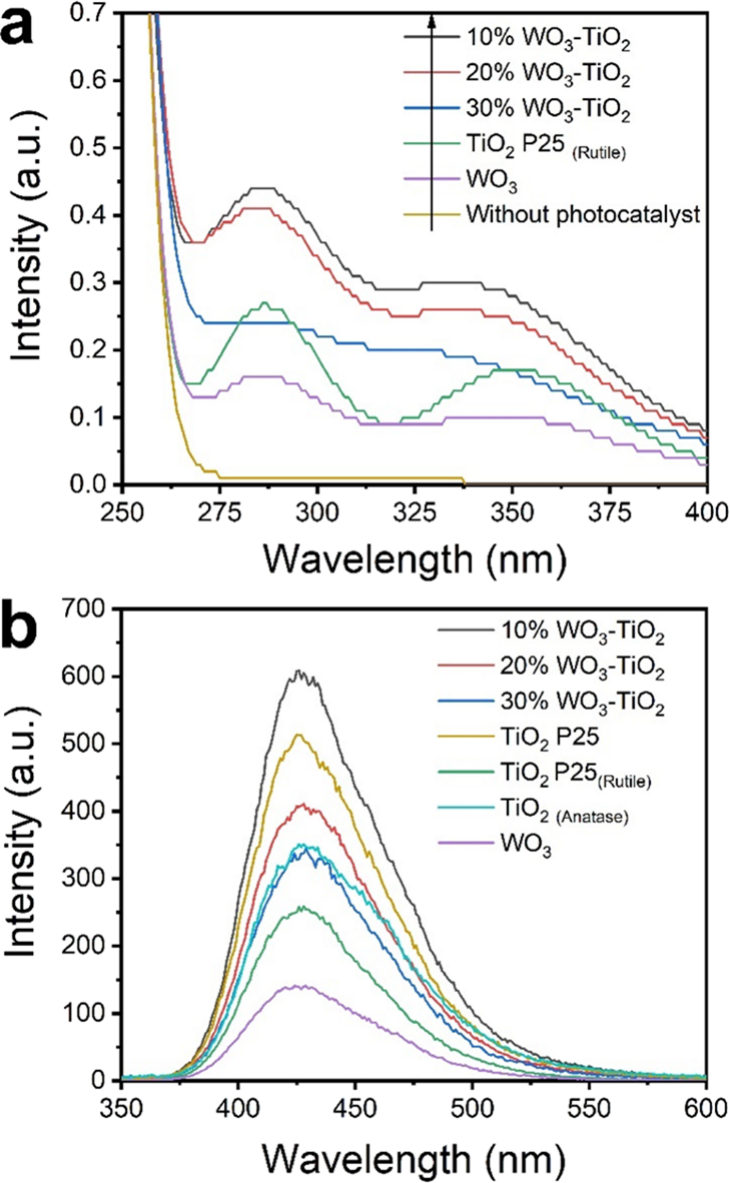
(a) Fluorescence spectra of KI solutions with various photocatalysts under near-UV illumination. (b) Fluorescence spectra of 2-hydroxy-terephthalic acid in the presence of various photocatalysts (315 nm excitation).

Figure 6a depicts the fluorescence spectra of heterojunctions and pure oxides under near-UV illumination. All photocatalysts showed the characteristic tri-iodine ion () peaks, indicating that holes (h^+^) are formed at the valence band of both WO_3_ and TiO_2_. The spectrum of both 10%WO_3_-TiO_2_ and 20%WO_3_-TiO_2_ shows the highest intensity, which is proportional to the h^+^ density. Additionally, the abundance of ^·^OH radicals was evaluated via the production of 2-hydroxy-terephthalic acid using fluorescence spectroscopy (Figure 6b).^30^ The 10%WO_3_-TiO_2_ photocatalyst showed the highest fluorescence signal, indicating that it not only exhibits a high h*^+^* density but also yields the highest production of ^·^OH radicals. These results are consistent with MO degradation under near-UV and visible light, confirming a degradation pathway mainly due to ^·^OH radicals.

### 3.5 Activation Mechanism under Near-UV and Visible Light

We are now in the position to propose an activation mechanism for the TiO_2(Anatase)_/WO_3_/TiO_2(Rutile)_ heterojunctions under UV light where the improved performance is attributed to the coexistence of all three materials. Under UV irradiation, all three phases contribute to e^–^–h^+^ pair production; however, generated electrons in the conduction band (CB) of anatase can be easily transferred to the CB of both rutile and WO_3_ because of its less negative redox potential in comparison with anatase. Similarly, the generated h^+^ in the valence band (VB) of WO_3_ can be transferred to the VB of anatase, resulting in reduced charge recombination and improved production of hydroxyl radicals. In addition, the generated h^+^ in all phases have suitable redox potentials to produce hydroxyl radicals that can oxidize adsorbed dye molecules, as specified in eqs 6–10 in the reaction mechanism, producing, through several intermediates, CO_2_ and H_2_O (eq 10).

Superoxide anions are produced by the photogenerated electrons and oxygen molecules that later form peroxide radicals when interacting with protons. These peroxyl radicals also interact with organic compounds to form intermediates, CO_2_, and H_2_O (eqs 11–13). Additionally, peroxyl radicals can form hydrogen peroxide and oxygen molecules (eq 14) by interacting with protons and superoxide anions. The produced hydrogen peroxide can be later broken down into ^·^OH radicals by UV light (eq 15).
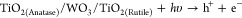
6
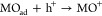
7
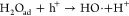
8
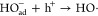
9
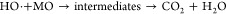
10
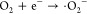
11
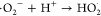
12
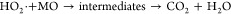
13
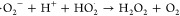
14
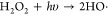
15

**Figure 7:**
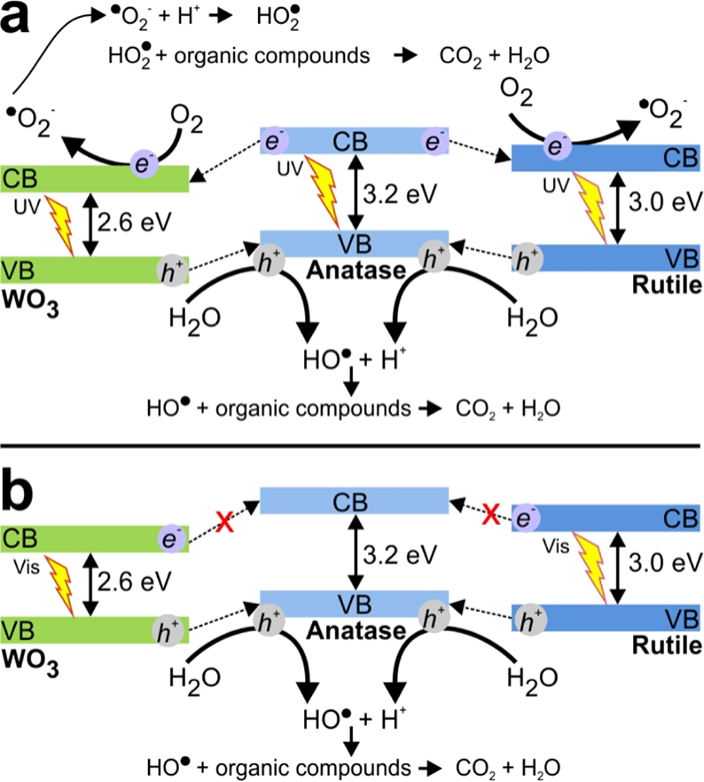
Proposed activation mechanism for TiO_2(Anatase)_/WO_3_/TiO_2(Rutile)_ double-heterojunction photocatalysts under (a) near-UV and (b) visible light irradiation.

It should be emphasized that all three WO_3_-TiO_2_ heterojunctions also displayed photocatalytic activity under visible light. In this case, the activation mechanism (Figure 7b) considers that TiO_2_ rutile and monoclinic WO_3_ are activated upon visible light irradiation, generating e^–^–h^+^ pairs. The h^+^ generated in the VB of WO_3_ and rutile produce hydroxyl radicals that can be transferred to the anatase VB. As a result, a reduced charge recombination is obtained with a subsequent increase in ^·^OH radical formation, as shown in eq 8. In this situation, the generated electrons in the conduction bands of WO_3_ and rutile are not transferred to anatase because of their more positive redox potential than those of anatase.

## 4 Conclusions

Double-heterojunction TiO_2(Anatase)_/WO_3_/TiO_2(Rutile)_ photocatalysts were synthetized by a simple sol–gel method. The 10%WO_3_-TiO_2_ and 20%WO_3_-TiO_2_ composites showed the largest synergistic effect observed during the degradation of methyl orange under UV and visible light. The best MO degradation was achieved with 10 wt % WO_3_-TiO_2_, and this photocatalyst exhibited a relatively large *E*_g_, large particle size, and low surface area; however, it exhibited the highest hole density and ^·^OH production, resulting in enhanced photocatalytic activity. The synergistic effect was explained by considering the formation of a double heterojunction between anatase, rutile, and WO_3_. Our study shows that tuning the materials’ content and crystal phase during the catalyst production can result in significant changes in the photocatalytic activity.

## Supporting Information Available

Derivation of the first-order model, emission spectra of the light sources, specific surface areas of all photocatalysts, electron microscopy images, and data fitting of kinetic models (PDF)

## References

[ref1] Pinedo A.; López M.; Leyva E.; Zermeño B.; Serrano B.; Moctezuma E. Photocatalytic Decomposition of Metoprolol and Its Intermediate Organic Reaction Products: Kinetics and Degradation Pathway. Int. J. Chem. React. Eng. 2016, 14, 809–820. 10.1515/ijcre-2015-0132.

[ref2] Moctezuma E.; Leyva E.; López M.; Pinedo A.; Zermeño B.; Serrano B. Photocatalytic Degradation of Metoprolol Tartrate. Top. Catal. 2013, 56, 1875–1882. 10.1007/s11244-013-0119-x.

[ref3] Likodimos V.; Han C.; Pelaez M.; Kontos A. G.; Liu G.; Zhu D.; Liao S.; de la Cruz A. A.; O’Shea K.; Dunlop P. S. M.; Byrne J. A.; Dionysiou D. D.; Falaras P. Anion-Doped TiO_2_ Nanocatalysts for Water Purification under Visible Light. Ind. Eng. Chem. Res. 2013, 52, 13957–13964. 10.1021/ie3034575.

[ref4] Shaham-Waldmann N.; Paz Y. Away from TiO_2_: A Critical Minireview on the Developing of New Photocatalysts for Degradation of Contaminants in Water. Mater. Sci. Semicond. Process. 2016, 42, 72–80. 10.1016/j.mssp.2015.06.068.

[ref5] Pelaez M.; Nolan N. T.; Pillai S. C.; Seery M. K.; Falaras P.; Kontos A. G.; Dunlop P. S. M.; Hamilton J. W. J.; Byrne J. A.; O’Shea K.; Entezari M. H.; Dionysiou D. D. A Review on the Visible Light Active Titanium Dioxide Photocatalysts for Environmental Applications. Appl. Catal. B Environ. 2012, 125, 331–349. 10.1016/j.apcatb.2012.05.036.

[ref6] Truong Q. D.; Liu J.-Y.; Chung C.-C.; Ling Y.-C. Photocatalytic Reduction of CO_2_ on FeTiO_3_/TiO_2_ Photocatalyst. Catal. Commun. 2012, 19, 85–89. 10.1016/j.catcom.2011.12.025.

[ref7] Liu X.; Ye L.; Liu S.; Li Y.; Ji X. Photocatalytic Reduction of CO_2_ by ZnO Micro/Nanomaterials with Different Morphologies and Ratios of {0001} Facets. Sci. Rep. 2016, 6, 38474 10.1038/srep38474. 27922092 PMC5138834

[ref8] Sarkar A.; Gracia-Espino E.; Wågberg T.; Shchukarev A.; Mohl M.; Rautio A. R.; Pitkänen O.; Sharifi T.; Kordas K.; Mikkola J. P. Photocatalytic Reduction of CO_2_ with H_2_O over Modified TiO_2_ Nanofibers: Understanding the Reduction Pathway. Nano Res. 2016, 9, 1956–1968. 10.1007/s12274-016-1087-9.

[ref9] Rusinque B.; Escobedo S.; de Lasa H. Photocatalytic Hydrogen Production Under Near-UV Using Pd-Doped Mesoporous TiO_2_ and Ethanol as Organic Scavenger. Catalysts 2019, 9, 33 10.3390/catal9010033.

[ref10] Yousaf A. B.; Imran M.; Zaidi S. J.; Kasak P. Highly Efficient Photocatalytic Z-Scheme Hydrogen Production over Oxygen-Deficient WO_3-x_ Nanorods Supported Zn_0.3_Cd_0.7_S Heterostructure. Sci. Rep. 2017, 7, 6574 10.1038/s41598-017-06808-6. 28747786 PMC5529397

[ref11] Pérez-Larios A.; Hernández-Gordillo A.; Morales-Mendoza G.; Lartundo-Rojas L.; Mantilla Á.; Gómez R. Enhancing the H_2_ Evolution from Water-Methanol Solution Using Mn^2+^-Mn^3+^-Mn^4+^ Redox Species of Mn-Doped TiO_2_ Sol-Gel Photocatalysts. Catal. Today 2016, 266, 9–16. 10.1016/j.cattod.2015.12.029.

[ref12] Zhong X.; Jin M.; Dong H.; Liu L.; Wang L.; Yu H.; Leng S.; Zhuang G.; Li X.; Wang J. G. TiO_2_ Nanobelts with a Uniform Coating of G-C_3_N_4_ as a Highly Effective Heterostructure for Enhanced Photocatalytic Activities. J. Solid State Chem. 2014, 220, 54–59. 10.1016/j.jssc.2014.08.016.

[ref13] Daghrir R.; Drogui P.; Robert D. Modified TiO_2_ For Environmental Photocatalytic Applications: A Review. Ind. Eng. Chem. Res. 2013, 52, 3581–3599. 10.1021/ie303468t.

[ref14] Fagan R.; McCormack D. E.; Dionysiou D. D.; Pillai S. C. A Review of Solar and Visible Light Active TiO_2_ Photocatalysis for Treating Bacteria, Cyanotoxins and Contaminants of Emerging Concern. Mater. Sci. Semicond. Process. 2016, 42, 2–14. 10.1016/j.mssp.2015.07.052.

[ref15] Schneider J.; Matsuoka M.; Takeuchi M.; Zhang J.; Horiuchi Y.; Anpo M.; Bahnemann D. W. Understanding TiO_2_ Photocatalysis: Mechanisms and Materials. Chem. Rev. 2014, 114, 9919–9986. 10.1021/cr5001892. 25234429

[ref16] Zhang S. T.; Ruan Y. R.; Liu C.; Wang P.; Ma Y. Q. The Evolution of Structure, Chemical State and Photocatalytic Performance of α-Fe/FeTiO_3_/TiO_2_ with the Nitridation at Different Temperatures. Mater. Res. Bull. 2017, 95, 503–508. 10.1016/j.materresbull.2017.08.042.

[ref17] Ctibor P.; Pala Z.; Stengl V.; Musalek R. Photocatalytic Activity of Visible-Light-Active Iron-Doped Coatings Prepared by Plasma Spraying. Ceram. Int. 2014, 40, 2365–2372. 10.1016/j.ceramint.2013.08.007.

[ref18] Hu L.; Wang J.; Zhang J.; Zhang Q.; Liu Z. An N-Doped Anatase/Rutile TiO_2_ Hybrid from Low-Temperature Direct Nitridization: Enhanced Photoactivity under UV-/Visible-Light. RSC Adv. 2014, 4, 420 10.1039/c3ra44421j.

[ref19] Xie W.; Li R.; Xu Q. Enhanced Photocatalytic Activity of Se-Doped TiO_2_ under Visible Light Irradiation. Sci. Rep. 2018, 8, 8752 10.1038/s41598-018-27135-4. 29884870 PMC5993730

[ref20] Dozzi M. V.; Selli E. Doping TiO_2_ with P-Block Elements: Effects on Photocatalytic Activity. J. Photochem. Photobiol. C 2013, 14, 13–28. 10.1016/j.jphotochemrev.2012.09.002.

[ref21] Yu C.; Zhou W.; Yu J. C.; Liu H.; Wei L. Design and Fabrication of Heterojunction Photocatalysts for Energy Conversion and Pollutant Degradation. Chin. J. Catal. 2014, 35, 1609–1618. 10.1016/S1872-2067(14)60170-4.

[ref22] Huo Y.; Chen X.; Zhang J.; Pan G.; Jia J.; Li H. Ordered Macroporous Bi_2_O_3_/TiO_2_ Film Coated on a Rotating Disk with Enhanced Photocatalytic Activity under Visible Irradiation. Appl. Catal. B Environ. 2014, 148-149, 550–556. 10.1016/j.apcatb.2013.11.040.

[ref23] Zhao Z. J.; Hwang S. H.; Jeon S.; Hwang B.; Jung J. Y.; Lee J.; Park S. H.; Jeong J. H. Three-Dimensional Plasmonic Ag/TiO_2_ Nanocomposite Architectures on Flexible Substrates for Visible-Light Photocatalytic Activity. Sci. Rep. 2017, 7, 8915 10.1038/s41598-017-09401-z. 28827643 PMC5566718

[ref24] Park H.; Park Y.; Kim W.; Choi W. Surface Modification of TiO_2_ Photocatalyst for Environmental Applications. J. Photochem. Photobiol. C 2013, 15, 1–20. 10.1016/j.jphotochemrev.2012.10.001.

[ref25] Sheikh F. A.; Appiah-Ntiamoah R.; Zargar A. M.; Chandradass J.; Chung W.-J.; Kim H. Photocatalytic Properties of Fe_2_O_3_-Modified Rutile TiO_2_ Nanofibers Formed by Electrospinning Technique. Mater. Chem. Phys. 2015, 172, 62–68. 10.1016/j.matchemphys.2015.12.060.

[ref26] Liu C.; Zhang L.; Liu R.; Gao Z.; Yang X.; Tu Z.; Yang F.; Ye Z.; Cui L.; Xu C.; Li Y. Hydrothermal Synthesis of N-Doped TiO_2_ Nanowires and N-Doped Graphene Heterostructures with Enhanced Photocatalytic Properties. J. Alloys Compd. 2016, 656, 24–32. 10.1016/j.jallcom.2015.09.211.

[ref27] Saien J.; Mesgari Z. Highly Efficient Visible-Light Photocatalyst of Nitrogen-Doped TiO_2_ Nanoparticles Sensitized by Hematoporphyrin. J. Mol. Catal. A: Chem. 2016, 414, 108–115. 10.1016/j.molcata.2015.12.027.

[ref28] Znad H.; Kawase Y. Synthesis and Characterization of S-Doped Degussa P25 with Application in Decolorization of Orange II Dye as a Model Substrate. J. Mol. Catal. A: Chem. 2009, 314, 55–62. 10.1016/j.molcata.2009.08.017.

[ref29] Sui Y.; Su C.; Yang X.; Hu J.; Lin X. Ag-AgBr Nanoparticles Loaded on TiO_2_ Nanofibers as an Efficient Heterostructured Photocatalyst Driven by Visible Light. J. Mol. Catal. A: Chem. 2015, 410, 226–234. 10.1016/j.molcata.2015.09.018.

[ref30] Rawal S. B.; Bera S.; Lee D.; Jang D.-J.; Lee W. I. Design of Visible-Light Photocatalysts by Coupling of Narrow Bandgap Semiconductors and TiO_2_: Effect of Their Relative Energy Band Positions on the Photocatalytic Efficiency. Catal. Sci. Technol. 2013, 3, 1822 10.1039/c3cy00004d.

[ref31] Sood S.; Mehta S. K.; Sinha A. S. K.; Kansal S. K. Bi _2_ O _3_ /TiO _2_ Heterostructures: Synthesis, Characterization and Their Application in Solar Light Mediated Photocatalyzed Degradation of an Antibiotic, Ofloxacin. Chem. Eng. J. 2016, 290, 45–52. 10.1016/j.cej.2016.01.017.

[ref32] Huang H.; Li D.; Lin Q.; Zhang W.; Shao Y.; Chen Y.; Sun M.; Fu X. Efficient Degradation of Benzene over LaVO_4_/TiO_2_ Nanocrystalline Heterojunction Photocatalyst under Visible Light Irradiation. Environ. Sci. Technol. 2009, 43, 4164–4168. 10.1021/es900393h. 19569346

[ref33] Lu Z.; Zeng L.; Song W.; Qin Z.; Zeng D.; Xie C. In Situ Synthesis of C-TiO_2_/g-C_3_N_4_ Heterojunction Nanocomposite as Highly Visible Light Active Photocatalyst Originated from Effective Interfacial Charge Transfer. Appl. Catal. B Environ. 2017, 202, 489–499. 10.1016/j.apcatb.2016.09.052.

[ref34] Yao Y.; Qin J.; Chen H.; Wei F.; Liu X.; Wang J.; Wang S. One-Pot Approach for Synthesis of N-Doped TiO_2_/ZnFe_2_O_4_ Hybrid as an Efficient Photocatalyst for Degradation of Aqueous Organic Pollutants. J. Hazard. Mater. 2015, 291, 28–37. 10.1016/j.jhazmat.2015.02.042. 25748999

[ref35] Lu B.; Ma N.; Wang Y.; Qiu Y.; Hu H.; Zhao J.; Liang D.; Xu S.; Li X.; Zhu Z.; Cui C. Visible-Light-Driven TiO_2_/Ag_3_PO_4_/GO Heterostructure Photocatalyst with Dual-Channel for Photo-Generated Charges Separation. J. Alloys Compd. 2015, 630, 163–171. 10.1016/j.jallcom.2015.01.008.

[ref36] Ke D.; Liu H.; Peng T.; Liu X.; Dai K. Preparation and Photocatalytic Activity of WO_3_/TiO_2_ Nanocomposite Particles. Mater. Lett. 2008, 62, 447–450. 10.1016/j.matlet.2007.05.060.

[ref37] Ismail M.; Bousselmi L.; Zahraa O. Photocatalytic Behavior of WO_3_-Loaded TiO_2_ Systems in the Oxidation of Salicylic Acid. J. Photochem. Photobiol., A 2011, 222, 314–322. 10.1016/j.jphotochem.2011.07.001.

[ref38] Gao L.; Gan W.; Qiu Z.; Zhan X.; Qiang T.; Li J. Preparation of Heterostructured WO_3_/TiO_2_ Catalysts from Wood Fibers and Its Versatile Photodegradation Abilities. Sci. Rep. 2017, 7, 1102 10.1038/s41598-017-01244-y. 28439084 PMC5430625

[ref39] Alves De Castro I.; Ariane De Oliveira J.; Cristina Paris E.; Regina Giraldi T.; Ribeiro C. Production of Heterostructured TiO_2_/WO_3_ Nanoparticulated Photocatalysts through a Simple One Pot Method. Ceram. Int. 2015, 41, 3502–3510. 10.1016/j.ceramint.2014.11.001.

[ref40] Huerta-Flores A. M.; Torres-Martínez L. M.; Moctezuma E.; Singh A. P.; Wickman B. Green Synthesis of Earth-Abundant Metal Sulfides (FeS_2_, CuS, and NiS_2_) and Their Use as Visible-Light Active Photocatalysts for H_2_ Generation and Dye Removal. J. Mater. Sci.: Mater. Electron. 2018, 29, 11613–11626. 10.1007/s10854-018-9259-x.

[ref41] Wu X.; Yin S.; Dong Q.; Guo C.; Li H.; Kimura T.; Sato T. Synthesis of High Visible Light Active Carbon Doped TiO_2_ Photocatalyst by a Facile Calcination Assisted Solvothermal Method. Appl. Catal. B Environ. 2013, 450 10.1016/j.apcatb.2013.05.052.

[ref42] Greczynski G.; Hultman L. X-Ray Photoelectron Spectroscopy: Towards Reliable Binding Energy Referencing. Prog. Mater. Sci. 2020, 100591 10.1016/j.pmatsci.2019.100591.

[ref43] Huerta-Flores A. M.; Mora-Hernández J. M.; Torres-Martínez L. M.; Moctezuma E.; Sánchez-Martínez D.; Zarazúa-Morín M. E.; Wickman B. Extended Visible Light Harvesting and Boosted Charge Carrier Dynamics in Heterostructured Zirconate–FeS_2_ Photocatalysts for Efficient Solar Water Splitting. J. Mater. Sci.: Mater. Electron. 2018, 29, 18957–18970. 10.1007/s10854-018-0019-8.

[ref44] Riboni F.; Bettini L. G.; Bahnemann D. W.; Selli E. WO_3_-TiO_2_ vs. TiO_2_ Photocatalysts: Effect of the W Precursor and Amount on the Photocatalytic Activity of Mixed Oxides. Catal. Today 2013, 209, 28–34. 10.1016/j.cattod.2013.01.008.

[ref45] Couselo N.; García Einschlag F. S.; Candal R. J.; Jobbágy M. Tungsten-Doped TiO_2_ vs Pure TiO_2_ Photocatalysts: Effects on Photobleaching Kinetics and Mechanism. J. Phys. Chem. C 2008, 1094 10.1021/jp0769781.

[ref46] Wang G.; Xu L.; Zhang J.; Yin T.; Han D. Enhanced Photocatalytic Activity of Powders (P25) via Calcination Treatment. Int. J. Photoenergy 2012, 2012, 1–9. 10.1155/2012/265760.

[ref47] Li Y.; Lv K.; Ho W.; Dong F.; Wu X.; Xia Y. Hybridization of Rutile TiO_2_ (RTiO_2_) with g-C_3_N_4_ Quantum Dots (CN QDs): An Efficient Visible-Light-Driven Z-Scheme Hybridized Photocatalyst. Appl. Catal. B Environ. 2017, 611 10.1016/j.apcatb.2016.09.055.

[ref48] Sánchez-Martínez D.; Gomez-Solis C.; Torres-Martinez L. M. CTAB-Assisted Ultrasonic Synthesis, Characterization and Photocatalytic Properties of WO_3_. Mater. Res. Bull. 2015, 165 10.1016/j.materresbull.2014.10.034.

[ref49] Ghasempour F.; Azimirad R.; Amini A.; Akhavan O. Visible Light Photoinactivation of Bacteria by Tungsten Oxide Nanostructures Formed on a Tungsten Foil. Appl. Surf. Sci. 2015, 55 10.1016/j.apsusc.2015.01.217.

[ref50] Kunyapat T.; Xu F.; Neate N.; Wang N.; De Sanctis A.; Russo S.; Zhang S.; Xia Y.; Zhu Y. Ce-Doped Bundled Ultrafine Diameter Tungsten Oxide Nanowires with Enhanced Electrochromic Performance. Nanoscale 2018, 4718 10.1039/c7nr08385h. 29464250

[ref51] Song H. Y.; Jiang H. F.; Liu X. Q.; Jiang Y. Z.; Meng G. Y. Preparation of WO_x_-TiO_2_ and the Photocatalytic Activity under Visible Irradiation. Key Eng. Mater. 2007, 336-338, 1979–1982. 10.4028/www.scientific.net/kem.336-338.1979.

[ref52] Baklanova I. V.; Krasil’nikov V. N.; Zhukov V. P.; Gyrdasova O. I.; Kuznetsov M. V.; Buldakova L. Y.; Yanchenko M. Y. Synthesis, Spectral, Optical and Photocatalytic Properties of Vanadium- and Carbon-Doped Titanium Dioxide with Three-Dimensional Architecture of Aggregates. J. Photochem. Photobiol., A 2016, 6 10.1016/j.jphotochem.2015.08.002.

[ref53] Lange F.; Schmelz H.; Knözinger H. An X-Ray Photoelectron Spectroscopy Study of Oxides of Arsenic Supported on TiO_2_. J. Electron Spectros. Relat. Phenomena 1991, 57, 307–315. 10.1016/0368-2048(91)80017-O.

[ref54] Liu X. W.; Yang Z. Z.; Pan F. S.; Gu L.; Yu Y. Anchoring Nitrogen-Doped TiO_2_Nanocrystals on Nitrogen-Doped 3D Graphene Frameworks for Enhanced Lithium Storage. Chem. - A Eur. J. 2017, 1757 10.1002/chem.201604603. 27922730

[ref55] Zhang T.; Wang L.; Su J.; Guo L. Branched Tungsten Oxide Nanorod Arrays Synthesized by Controlled Phase Transformation for Solar Water Oxidation. Chem CatChem 2016, 8, 2119–2127. 10.1002/cctc.201600267.

[ref56] Bertus L. M.; Faure C.; Danine A.; Labrugere C.; Campet G.; Rougier A.; Duta A. Synthesis and Characterization of WO_3_ Thin Films by Surfactant Assisted Spray Pyrolysis for Electrochromic Applications. Mater. Chem. Phys. 2013, 140, 49–59. 10.1016/j.matchemphys.2013.02.047.

[ref57] Yu H.; Guo J.; Wang C.; Zhang J.; Liu J.; Zhong X.; Dong G.; Diao X. High Performance in Electrochromic Amorphous WO_x_ Film with Long-Term Stability and Tunable Switching Times via Al/Li-Ions Intercalation/Deintercalation. Electrochim. Acta 2019, 644 10.1016/j.electacta.2019.06.129.

[ref58] Alov N. V. XPS Study of MoO_3_and WO_3_Oxide Surface Modification by Low-Energy Ar^+^ Ion Bombardment. Phys. Status Solidi C 2015, 263 10.1002/pssc.201400108.

[ref59] Vaez Z.; Javanbakht V. Synthesis, Characterization and Photocatalytic Activity of ZSM-5/ZnO Nanocomposite Modified by Ag Nanoparticles for Methyl Orange Degradation. J. Photochem. Photobiol., A 2020, 388, 112064 10.1016/j.jphotochem.2019.112064.

[ref60] Herrmann J.-M. Photocatalysis Fundamentals Revisited to Avoid Several Misconceptions. Appl. Catal. B Environ. 2010, 99, 461–468. 10.1016/j.apcatb.2010.05.012.

[ref61] Uddin M. J.; Islam M. A.; Haque S. A.; Hasan S.; Amin M. S. A.; Rahman M. M. Preparation of Nanostructured TiO_2_-Based Photocatalyst by Controlling the Calcining Temperature and PH. Int. Nano Lett. 2012, 2, 19 10.1186/2228-5326-2-19.

[ref62] Talebi S.; Chaibakhsh N.; Moradi-Shoeili Z. Application of Nanoscale ZnS/TiO_2_ Composite for Optimized Photocatalytic Decolorization of a Textile Dye. J. Appl. Res. Technol. 2017, 15, 378–385. 10.1016/j.jart.2017.03.007.

