# Supplementary material for: Nanoparticulate Double-Heterojunction Photocatalysts Comprising TiO2(Anatase)/WO3/TiO2(Rutile) with Enhanced Photocatalytic Activity toward the Degradation of Methyl Orange under Near-Ultraviolet and Visible Light
Source: ACS Omega. 2021 Apr 28;6(18):11840–8. doi: 10.1021/acsomega.0c06054 (PMC8154020; doi:10.1021/acsomega.0c06054)
Supplement: Supplementary file 1 — ao0c06054_si_001.pdf [file ao0c06054_si_001.pdf]

Supplementary Information

Nanoparticulate Double Heterojunction Photocatalysts Comprising $TiO_{2(Anatase)}/WO_3/TiO_{2(Rutile)}$ with Enhanced Photocatalytic Activity towards the Degradation of Methyl Orange under near UV and Visible Light

José Alfonso Pinedo-Escobar[a,*], *Junpeng Fan*[b], *Edgar Moctezuma*[c], *Christian Gomez-Solís*[d], *Cristina Jared Carrillo Martinez*[a] *and Eduardo Gracia-Espino*[b,*]

[a] Unidad Académica de Ciencias Químicas, Universidad Autónoma de Zacatecas, Campus Universitario Siglo XXI, km. 6 Carr. Zacatecas-Guadalajara s/n Ejido La Escondida, 98160, Zacatecas, Zac., México.

[b] Department of Physics, Umeå University, Umeå 90187, Sweden.

[c] Facultad de Ciencias Químicas, Universidad Autónoma de San Luis Potosí, Av. Manuel Nava #6, 78290, San Luis Potosí, S.L.P., México

[d] División de Ciencias e Ingeniería, Universidad de Guanajuato, 37150, León, Guanajuato, México.

*Corresponding authors: J.A.P.-E. (alfonso.pinedo@uaz.edu.mx), and E.G.-E. (eduardo.gracia@umu.se)

The First-Order model was derived from the Langmuir-Hinshelwood model that describes the reaction rate on photocatalyst surfaces according to equation R1,

$$r = k_{true} q_c \qquad \text{(R1)}$$

where q_c is given by,

$$q_c = \frac{q_m k_2 C}{1 + k_2 C} \qquad \text{(R2)}$$

by substituting R2 in R1, we get,

$$r = k_{true} \left(\frac{q_m k_2 C}{1 + k_2} \right) \qquad \text{(R3)}$$

r = Reaction rate on the photocatalyst surface; mol $g^{-1}min^{-1}$

k_{true} = True constant reaction rate on the photocatalyst surface; min^{-1}

k_2 = Adsorption constant; L mol^{-1}

q_c = Amount of compound adsorbed on the photocatalyst surface; mol g^{-1}

q_m = Maximum amount of compound adsorbed on the photocatalyst surface; mol g^{-1}

C = Concentration of the compound at adsorption equilibrium; mol L^{-1}

k_1 is then defined as:

$$k_1 = k_{true} q_m \quad \text{(R4)}$$

Now substituting R4 in R3, the reaction rate is now given by:

$$r = \frac{k_1 k_2 C}{1 + k_2 C} \qquad \text{(R5)}$$

Equation R5 is the same as equation #2 in the main manuscript. At very low concentration the term k_2C is much smaller than 1, making the *reaction rate being apparently of the first degree.*

$$r_{app} = \left(\frac{w}{V}\right) r \approx \left(\frac{w}{V}\right) k_1 k_2 C \quad \text{(R6)}$$

where:

r_{app} = apparent reaction rate; mol $L^{-1}min^{-1}$

w = photocatalyst mass; g

V = reaction volume; L

We now define k_{app} as:

$$k_{app} = \left(\frac{w}{V}\right) k_1 k_2 = \left(\frac{w}{V}\right) k_{true} q_m k_2 \quad \text{(R7)}$$

From equation R7, we can see that k_{app} is proportional to the photocatalyst mass, true constant reaction rate, maximum amount of model compound adsorbed on the photocatalyst surface, and adsorption constant, and inversely proportional to the volume of the reaction.

Finally, we can substitute R7 in R6:

$$r_{app} \approx k_{app} C \quad \text{(R8)}$$

Equation R8 is the same as equation #3 in the main manuscript, which after integration becomes,

$$ln\left(\frac{C_0}{C}\right) = k_{app} t \quad \text{(R9)}$$

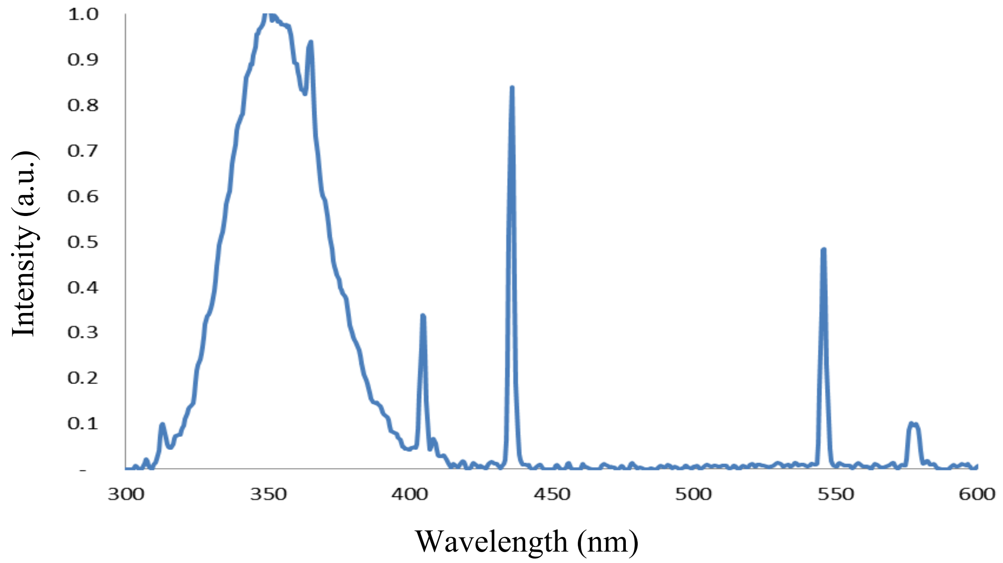

Figure S1. Emission spectrum of the near-UV light lamp T-15 L Cole-Parmer (15 Watts, λ_{max}= 365 nm)

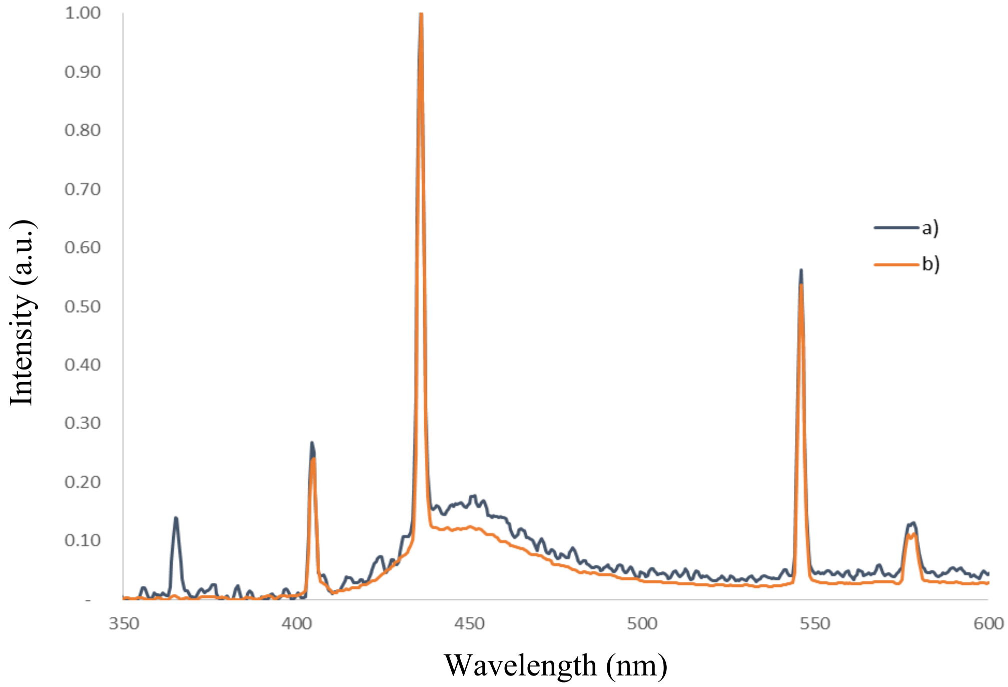

Figure S2. Emission spectrum of the visible light lamp FL15AQ Lumiaction (15 Watts, λ_{max}=436 nm) (a) without UV filter and (b) with UV filter

Table S1. Specific surface area of selected photocatalysts as obtained from the BET method.

**Photocatalysts**	**Specific surface area (m^2 g^{-1})**
10%WO_3-TiO_2	26.2
20%WO_3-TiO_2	27.9
30%WO_3-TiO_2	29.3
TiO_2 P25	44.1
TiO_2 P25$_{(rutile)}$	9.1
WO_3	5.0

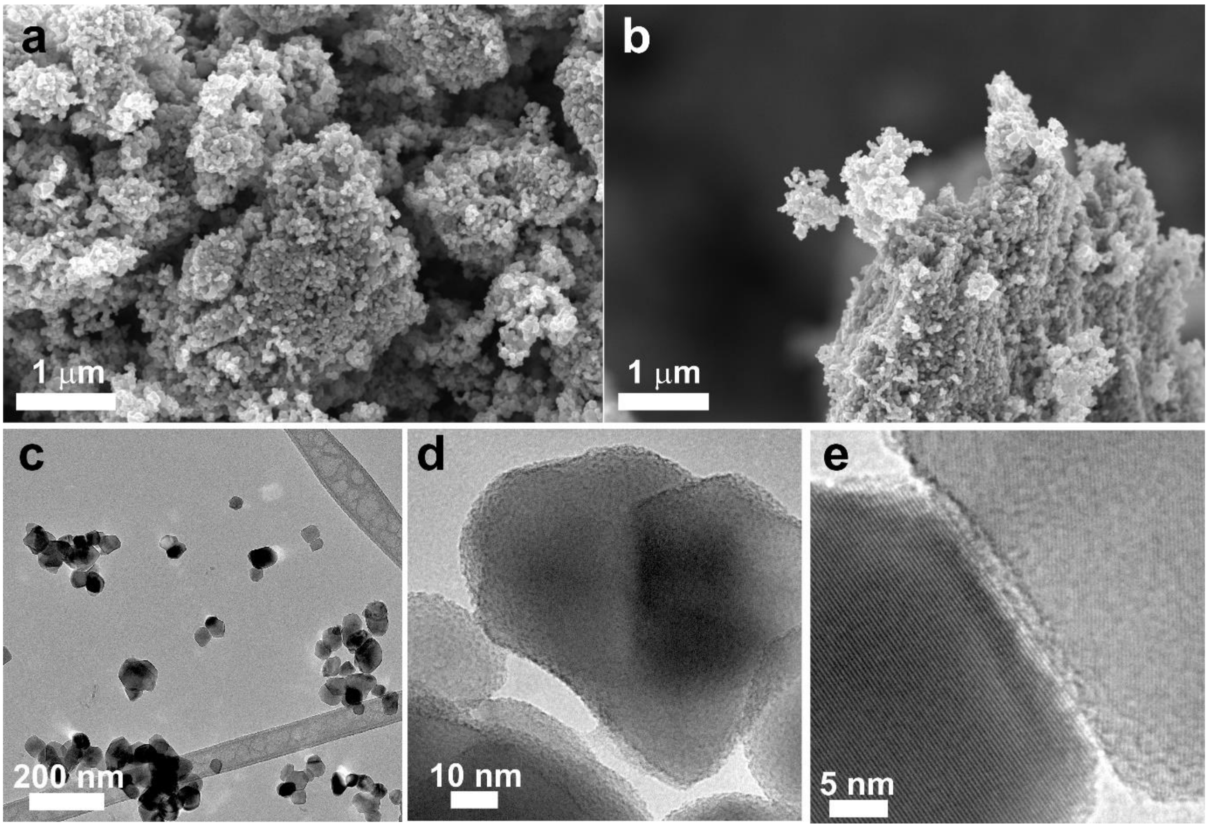

Figure S3. (a-b) SEM and (c-e) TEM micrographs of 10%WO_3-TiO_2

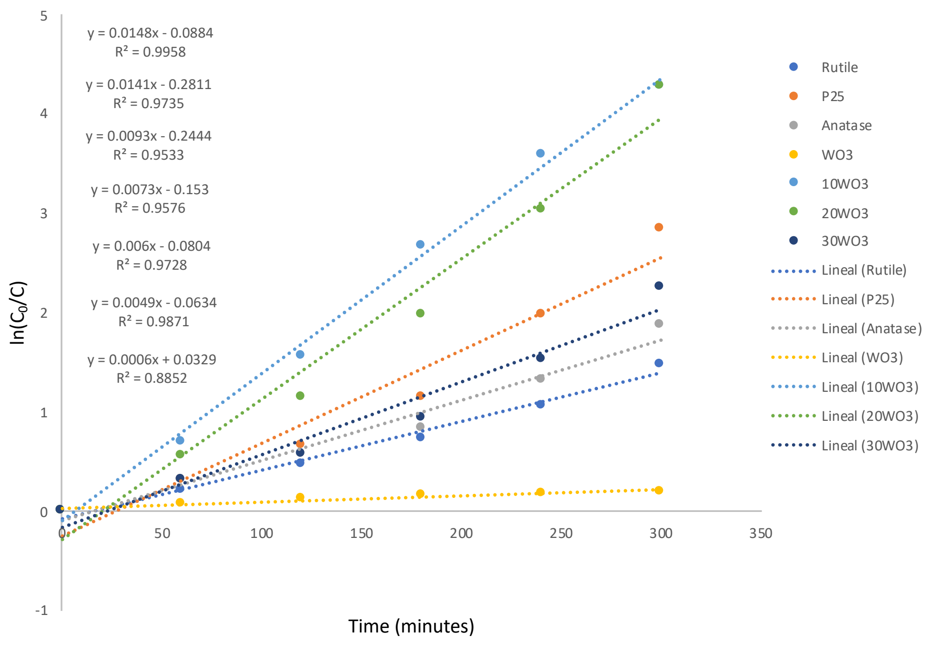

Figure S4. Kinetic fitting to First-Order model (20 ppm of MO degradation under near-UV light)

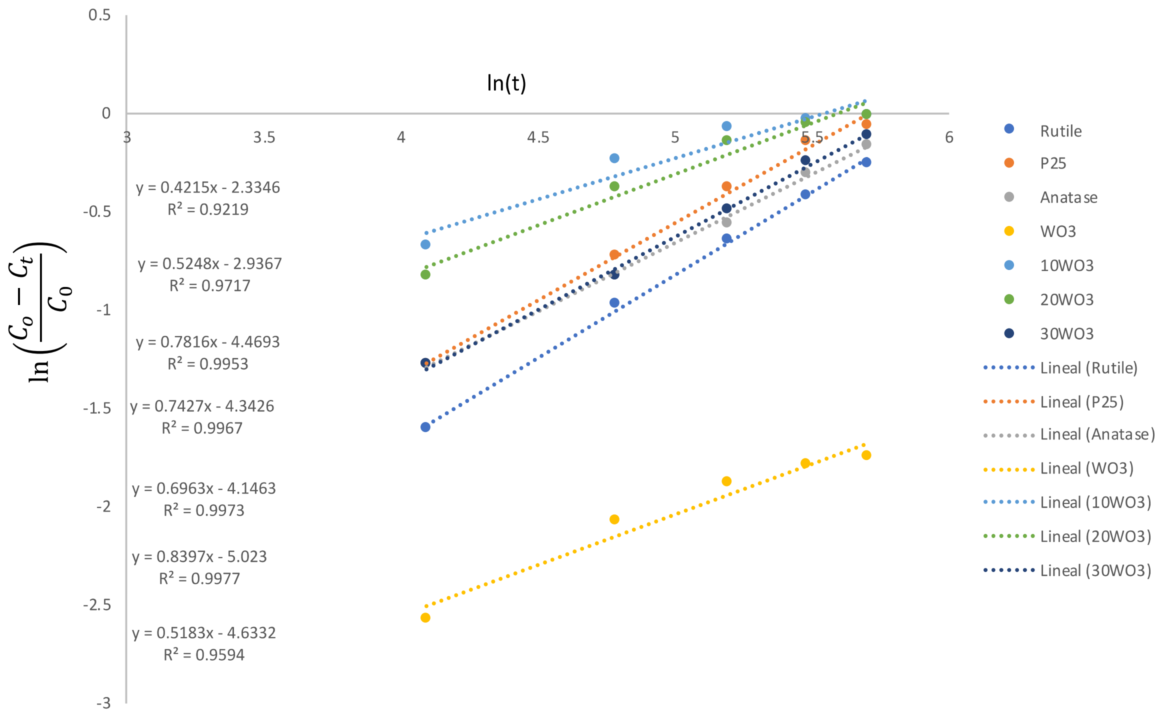

Figure S5. Kinetic fitting to Modified Freundlich model (20 ppm of MO degradation under near-UV light)
